# Flaxseed Enriched Pasta—Chemical Composition and Cooking Quality

**DOI:** 10.3390/foods9040404

**Published:** 2020-04-01

**Authors:** Piotr Zarzycki, Emilia Sykut-Domańska, Aldona Sobota, Dorota Teterycz, Ada Krawęcka, Agata Blicharz-Kania, Dariusz Andrejko, Beata Zdybel

**Affiliations:** 1 Division of Engineering and Cereals Technology, Department of Plant Food Technology and Gastronomy, University of Life Sciences, Skromna 8 Street, 20-704 Lublin, Poland; piotr.zarzycki@up.lublin.pl (P.Z.); aldona.sobota@up.lublin.pl (A.S.); dorota.teterycz@up.lublin.pl (D.T.); ada.krawecka@gmail.com (A.K.); 2 Department of Biological Bases of Food and Feed Technologies, Faculty of Production Engineering, University of Life Sciences, Głęboka 28 Street, 20-612 Lublin, Poland; agata.kania@up.lublin.pl (A.B.-K.); dariusz.andrejko@up.lublin.pl (D.A.); beata.zdybel@up.lublin.pl (B.Z.)

**Keywords:** flaxseed flour, flaxseed cake, pasta, chemical composition, cooking quality

## Abstract

Pasta production is a good opportunity for product innovation in different forms. The aim of this work was to assess the use of flaxseed components for pasta production. We examined the chemical composition and cooking quality, at different contents of flaxseed flour (FF) and flaxseed cake (FC), added for pasta processing. The analysis showed that the addition of flaxseed components to the dough caused a substantial difference in the International Commission on Illumination color model (CIE) parameter, compared to control samples. The samples of pasta with FF and FC were darker, redder, and less yellow than the control. The minimum cooking time for the enriched pasta was longer than that for the control pasta, although cooking losses were lower. The increasing content of flaxseed components did not significantly change the weight and volume increase index. The enrichment of pasta with 23% FF and 17% FC yielded good quality pasta. The results of the chemical composition of the flaxseed-enriched pasta indicate considerably enhanced nutritional quality, particularly the levels of protein, fat, and dietary fiber of the pasta, without affecting its quality. Moreover, flaxseed cake can be an important source of nutritional ingredients for pasta production, although it is a by-product of the oil cold pressing technology.

## 1. Introduction 

Pasta production is a good opportunity for product innovation in different forms [1,2]. The food technologists are interested in using flaxseed (*Linum usitatissimum*) in food production because of its health effects [3,4,5]. Cereal products can be prepared by fortification with different flaxseed forms [6]. The structure can affect the release of bioactive constituents of flaxseed at the target site of the digestive system. Flaxseeds must be milled or ground to access their full nutritional value [7]. Flaxseed is a rich source of alpha linolenic acid (ALA) which represents more than half of total fat in flaxseed. Imran et al. [8] found that raw flaxseed meal was characterized by the highest oxidative stability. They showed that extruded flaxseed meal can be used for production of health-enhancing products due to its long-term stability. The anti-nutritional factors that limit the use of flaxseed are cyanogenic glycosides, which release toxic hydrogen cyanide upon hydrolysis [9]. Udousoro and Etuk [10] reported that the heating process is very effective in the reduction of the content of anti-nutrients present in the foodstuff, thus ensuring safe consumption.

According to the research of Bhise et al. [11], a 10% addition of flaxseed flour into extruded noodles improved protein content. The influence of a higher content of flaxseed flour gave negative effects on physical and cooking features of products [11]. Flaxseed contains approximately 25–28% of total fiber, and the major fiber fractions are cellulose, mucilage gums, and lignin [12]. This component is also rich in lignans and secoisolariciresinol diglucoside (SDG), which provides additional health benefits [13]. A daily intake of 100 g pasta with 20% of flaxseed completely satisfies the daily needs of ω-3 essential fatty acids (5.9 g/100 g) recommended by the International Society for the Study of Fatty Acids and Lipids (ISSFAL) [14].

However, as flaxseed is primarily used for decoration and texture in baked products, the physicochemical properties of dried pasta manufactured with partial replacement of semolina by flaxseed flour and flaxseed cake is still little known. In this context, the objectives of the present study were to investigate the optimization levels of flaxseed components to improve the nutritional and sensory value of the product and to investigate the nutritional value of pasta manufactured with partial replacement of wheat flour by flaxseed cake and flaxseed flour.

## 2. Materials and Methods

### 2.1. Materials

Semolina (*Triticum durum* L.) was purchased from Julia Malom (Kunszállás, Hungary) and flaxseed flour was bought from Niedźwiady Mill (Niedźwiady, Poland). Flaxseed cake was prepared in a cold pressing process carried out using a screw press (DUO, Farmet, Czech Republic) at a capacity 18–25 kg h^−1^, screw speed 1500 rpm, dyes 10 mm, and barrel temperature 50 °C. The flaxseed cake was ground in an impact crusher (sieve diameter 5.0 mm).

### 2.2. Pasta Processing Conditions

Twelve pasta formulations were prepared using different semolina, flaxseed flour (FF), and flaxseed cake (FC) levels. A semi-technical laboratory scale pasta extruder MAC 30S-Lab (Ital Past, Italy) was used to produce pasta with flaxseed flour and flaxseed cake into a ribbon short-form cut in precise screw speed (45 rpm), cylinder (29 °C) and teflon dye (40 °C) temperature conditions. Flaxseed components were added in amounts of 5%, 9%, 13%, 17%, 20%, and 23% to replace the semolina (Table 1). All formulations were optimized to 31% moisture content and mixed for 15 min under atmospheric pressure. The produced pasta was pre-dried, placed on sieves, and dried in a static dryers EAC30-LAB (ItalPast, Italy) at low profile temperature (from 55 to 35 °C) in a 7 h drying cycle (75% to 55% of relative humidity). The dried pasta was stored in plastic bags at 4 °C until assessment. All pasta formulations were produced in duplicate.

### 2.3. The Color of Pasta

The color of pasta samples was measured using a colorimeter (X-Rite 8200, Inc., Grand Rapids, MI, USA) with a standard light source (D65), standard colorimetric observer (10°), and a 12.3 mm diameter hole. White and black calibration references were applied to standardize the instrument before analysis. The following CIE parameters were recorded: L* (lightness, indicates the level of light 100 or dark 0), a* (−a* = indicates greenness, +a* = indicates redness), and b* values (−b* = more blue, +b* = more yellow). The measurements were performed repeatedly 15 times per sample.

The total color difference (∆E*) as an overall measure between the treated and control sample was calculated with Equation (1).

(1) $$\Delta E^{*}=\sqrt{{\left(L_{c}^{*}-L_{i}^{*}\right)}^{2}+{\left(a_{c}^{*}-a_{i}^{*}\right)}^{2}+{\left(b_{c}^{*}-b_{i}^{*}\right)}^{2}}$$

where:

$L_{c}^{*},\;a_{c}^{*},\;b_{c}^{*}$-references (color parameters of the control sample),
$L_{i}^{*},\;a_{i}^{*},\;b_{i}^{*}$-olor parameters of the pasta sample.

The total color difference (∆E*) is usually classified as a trace level (0–0.5), slight (0.5–1.5), noticeable (1.5–3.0), appreciable (3.0–6.0), large (6.0–12.0), and obvious difference (>12.0).

The whiteness index (***WI***) of the pasta sample was also calculated according to the Equation (2).

(2) $$WI=100-{\left({\left(100-L^{*}\right)}^{2}+a^{2}+b^{2}\right)}^{0,5}$$

### 2.4. Cooking Quality and Firmness of Pasta

The minimum cooking time (MCT) of the pasta was determined as in Sobota et al. [15]. The disappearance of the white central core of the pasta samples squeezed between two plates of glass was indicative of the minimum cooking time. First, 100 g of pasta samples were put into 1000 mL boiling distilled water. Every 15 s, a pasta sample was tested to determine the MCT, until the white core could no longer be seen. Next, each pasta sample was cooked according to the determined cooking time; the pasta was strained, cooled, and weighed. Each time, dry matter losses (cooking losses) during the cooking were determined. The level of cooking loss was determined by AACC Method 66–50.01. The weight increase index (**WII**) and the volume increase index (**VII**) was calculated according to Sobota et al. [15]. The determinations of all cooking quality properties were made in three replications.

### 2.5. Firmness of Pasta

A 50 g sample of pasta was boiled in 600 mL of distilled water for the minimum cooking time determined previously. Next, it was drained and rinsed for 30 s with 100 mL of distilled water. The determination of firmness (maximum force required to cut the pasta) was made using a Zwick/Roell Z0.5 testing machine (maximum force 500 N). The speed of the head movement was constant (10 mm·s^−1^). Cutting tests were performed using a Warner–Bratzler flat knife. The measurements were carried out in ten replications for each sample. Based on the analysis, the force required to destroy the sample was determined using the test Xpert II software.

### 2.6. Chemical Composition of Raw Material and Pasta

Each pasta sample was analyzed according to AACC and AOAC methods [16,17]. The following parameters were determined: moisture AACC 44–15A, protein AACC 46–08, fat AACC 30–26, and ash AACC 08–01. The protein content was determined using a KjeltecTM8400 machine with the ASN 3100 application. The distillation was carried out in an automatic device Kjeltec Auto (Tecator) (N% × 5.7). The total fat was determined by continuous extraction using the SoxtecTM8000 machine with the AN 310 application. The total dietary fibre (TDF), soluble dietary fibre (SDF), and insoluble dietary fibre (IDF) was determined with the enzymatic method (AOAC 991.43; AACC 32–07; AACC 32–21; AOAC 985.29; AACC 32–05) using a Megazyme assay kit (Bray Co. Wicklor, Ireland). The available carbohydrate was calculated by the difference: 100–fat (%)–protein (%)–ash (%)–moisture (%)–TDF (%). The energy value (kcal/100g) was calculated using a modified Atwater factor (protein–4 kcal, carbohydrate–4 kcal, fat–9 kcal, TDF–2 kcal). All chemical tests were done in three replications.

### 2.7. Statistical Analysis

The data were subjected to statistical analysis (SAS ver. 9.2, Cary, NC, USA). Mean values, standard deviations, and significance of differences between mean values (Duncan test, *p* ≤ 0.05) were determined.

## 3. Results and Discussion

### 3.1. Pasta Processing

Flaxseed components create challenges for food producers due to their specific physical and nutritional features. The increasing content of flaxseed flour and cake in the enriched pasta resulted in lower pressure values, compared to control pasta (Table 1). The dough was pushed through a dye under diminished pressure from 12 MPa for the control pasta to 0.8 MPa (FC23) and 0.82 MPa (FF23). The lower pressure conditions yielded a reduced line output from 23kg·h^−1^ (CON) to 21.5 kg·h^−1^ (FF23) and 21.3 kg·h^−1^ (FC23).

### 3.2. Color Parameters

The color of pasta appeared to be changed by the factors analyzed in this study (Table 2). Statistically significant differences were observed between the control sample and samples with flaxseed components according to the CIE parameters. The most noticeable change was observed in the lightness (L*) of pasta enriched with 23% of flaxseed flour (FF23) compared to the control sample (Table 2). Parameter L* for products fortified with the flaxseed component was 37.4 and 41.4 for FF23 and FC23, respectively, and 61.1 for the CON sample. Biernacka et al. [18] found that the lightness (L*) of fifteen types of traditional pasta made by different producers ranged from 44.5 to 62.9. There was difference in L* values between spaghetti made with common and durum wheat flour. It was found that a*, which is associated with redness, diminished with the increasing content of the flaxseed component. An increase in the redness parameter was accompanied by the increase in the ash content [18]. Our results are in agreement with others [18,19]; the redness of the flaxseed-enriched pasta was higher than that of the control sample. The increasing addition of the flaxseed components from 5% to 23% diminished pasta yellowness compared to that of the control sample, i.e., from 20.5 (CON) to 4.1 (FF23) and 3.1 (FC23). These results are consistent with the report by Sinha and Manthey [19] who stated that flaxseed-free pasta was brighter and had weaker red color and stronger yellow compared to 5% ground flaxseed enriched pasta. The addition of flaxseed from 15% to 30% for pasta resulted in a stronger red color [20]. Although the natural carotenoid or xanthophyll pigments determine the color of traditional pasta, the concentrations of carotenoids in flaxseed differ from those in semolina [21,22,23]. This is responsible for the less yellow color of pasta supplemented with flaxseed components compared to semolina pasta.

### 3.3. Cooking Quality

Both the flaxseed flour and flaxseed cake influenced the minimum cooking time. An increase in the share of the flaxseed components in the products from 0% to 23% resulted in a statistically significant increase in the minimum cooking time, compared to the control sample (Table 3). The minimum cooking time of the flaxseed flour-enriched pasta ranged from 6 min (FF5) to 7 min (FF23) and from 6 min (FC5) to 6.15 min (FC23) for the flaxseed cake pasta. The physical disruption of the gluten matrix occurring when non-traditional ingredients are present in pasta can facilitate diffusion of water and reduce the cooking time of the product [24].

The addition of the flaxseed flour and flaxseed cake to replace the semolina induced a statistically significant decrease in the cooking losses, compared to the control sample (Table 3). The cooking loss values ranged from 5.42% d.b. (FF5) to 3.99% d.b. (FF23) for the pasta made of the flaxseed flour, and from 5.25% d.b. (FC5) to 4.44% d.b. (FC23) for pasta made of the flaxseed cake. Flaxseeds are source of lignans. Its high viscosity limits migration of dry matter components, e.g., starch, during cooking of pasta. The cooking loss of pasta enriched with flaxseed flour is lower compared to pasta enriched with flaxseed cake. Additionally, the flaxseed flour granulation was lower compared to flaxseed cake. This could explain the physical disruption of the gluten matrix. The cooking time of pasta with flaxseed flour was longer than cooking time of pasta with flaxseed cake, suggesting that the addition of flaxseed flour causes lower physical disruption of the gluten matrix. The cooking loss (CL) of fifteen types of traditional pasta made by different producers ranged from 4.76% to 6.55% [18]. It was observed that higher enrichment of pasta with reconstituted flaxseed (30% compared to 15%) gave higher cooking losses [20]. The authors claim that disruption of the gluten network makes for starch swelling and amylose leaching. These results are contradictory to those obtained by Manthey and others [25], who reported increased water absorption for 15% flaxseed enriched fresh pasta compared to flaxseed-free pasta. Physical properties of flaxseed dietary fiber can counterbalance the effect of starch dilution, which absorbs water, swells, and tends to solubilize during cooking. The weak wheat gluten network influences lost in the cooking water of amylose molecules [26]. Therefore, the addition of flaxseed for pasta production can decrease the water absorption index during cooking, which can be attributed to starch dilution. Insoluble dietary fiber competes with starch for water. WII is higher for pasta enriched with flaxseed components. Macaroni enriched with 15% of ground flaxseed (dried at 40 or 70 °C) compared to traditional pasta has 6% lower cooked weight [27]. Complexes of lipid and amylose characterized by low water solubility decide about the lower cooking losses.

The capacity of pasta to absorb water during cooking determined the value of the weight increase index WII of the products after the cooking. The value of the weight increase index of the flaxseed-enriched pasta did not differ statistically significantly from the control sample except for the FF23 probe (Table 3). The values of the index were 2.18 for the 5% addition of the flaxseed flour and 2.17 for the 5% addition of the flaxseed cake. The weight increase index of fifteen types of traditional pasta made by different producers ranged from 2.62 to 3.00 [18]. The same tendency was observed for the volume increase index of the flaxseed-enriched pasta. However, in this case, the samples with the addition of the flaxseed cake above 13% exhibited a statistically significantly higher volume index than the control sample.

### 3.4. Texture Parameters

An indirect indicator of pasta hardness is the pasta cutting force. Our results show that the addition of the flaxseed components decreased statistically significant the cutting force, compared to the control pasta (Table 4). The addition of the flaxseed flour gave statistically different cutting force values, compared to the control sample (Table 4). The cutting force ranged from 0.94 N (FF5) to 0.92 N (FF23) for the flaxseed flour addition and from 1.03 N (FC5) to 0.89 N (FC23) for the flaxseed cake addition. The decline in firmness with the increasing addition of the flaxseed components is related to weaker gluten matrices. The weak gluten matrices determined the high weight and volume increase index (Table 3), which is reflected in the firmness of pasta.

### 3.5. Chemical Composition and Nutritional Value

The addition of the flaxseed components exerted a level-dependent effect (*p* < 0.05) on the chemical composition of the pasta formulations (Table 5). The flaxseed components enhanced the nutritional value and kept the overall acceptability of products. Our results are consistent with earlier research [28]. The ash, protein, fat, and dietary fiber contents in the flaxseed pasta were higher than in the control (Table 5), which could be attributed to the fact that flaxseed is far higher in mineral, fat, protein, and fiber content than semolina durum. The addition of the flaxseed components decreased (*p* < 0.05) carbohydrate contents but increased (*p* < 0.05) the levels of ash, protein, lipid, and dietary fiber due to the flaxseed compositions. The flaxseed components exhibit low carbohydrate levels and increased protein, lipid, and ash contents (Table 5). The semolina counterpart contains increased amounts of carbohydrate and is low in protein, lipid, and ash. Pasta obtained from semolina with flaxseed cake was characterized by higher ash content than the pasta produced with flaxseed flour. The ash content in the pasta ranged from 1.11% (sample FF5) to 2.01% (sample FC23). The same tendency as in the ash content was found for the protein content: higher protein content was determined in the flaxseed cake pasta (ranging from 14.14% FF5 to 18.06% FF23), compared with the flaxseed flour pasta (from 14% FF to 17.93% FF), although the differences were not statistically significant. The flaxseed enrichment influences fat content increase, especially anti-inflammatory fatty acids. The fat content in the pasta with the flaxseed component ranged from 0.38% (FC5) to 3.08% (FC23) and from 0.73% (FF5) to 3.5% (FF23) for the flaxseed cake and flour, respectively, and was statistically different from that in the CON sample. Biernacka et al. [18] found that the fat content in commercially available pasta samples was similar, i.e., approximately 0.25% except wholemeal pasta samples (0.56%). The levels of dietary fiber fractions were higher in the flaxseed-enriched pasta than in the control (Table 5). The increase in dietary fiber was higher in the flaxseed cake pasta compared to that in the flaxseed flour pasta. The increase in IDF was greater than 300% for the flaxseed flour and cake addition, compared to the control sample. The same tendency was observed for the soluble dietary fiber fraction: pasta with 23% of the flaxseed flour and flaxseed cake had over 200% higher content of soluble dietary fiber, compared to the control sample. Enrichment of pasta with the flaxseed component resulted in reduced carbohydrate content, compared to the control. Masoodi and Bashir [29] showed that biscuits with flaxseed flour had lower carbohydrate content than the control sample. The addition of the nutrient-dense flaxseed components did not significantly change the energy value of the pasta, compared to the control sample. The energy value of a 100 g portion of the semolina pasta was 345.8 kcal, compared to 348.7 kcal of the flaxseed flour pasta and 348.1 kcal of the flaxseed cake pasta. Consumption of the same portion of flaxseed-enriched pasta and semolina pasta provides more nutrient-dense ingredients than semolina pasta. Given the positive influence of dietary fiber on human health, flaxseed-enriched pasta can be assumed to have a positive, health-enhancing impact. Nevertheless, more research is required to address this issue.

## 4. Conclusions

This research demonstrates that flaxseed flour and cake could technically be used for the production of a functional pasta product. Although pasta with flaxseed differs in terms of sensory quality from the conventional pasta to which consumers are accustomed, it certainly is a healthier option. The flaxseed components greatly enhanced the nutritional qualities of the products. Both flaxseed flour and cake enhanced the protein, fat, and dietary fiber in pasta without affecting quality properties. The results of the chemical composition of the flaxseed cake pasta indicate that it may be regarded as an important source of nutrition ingredients for pasta production, although it is a by-product of the oil cold pressing technology.

## Figures and Tables

**Table 1:** Model of experiment and production parameters.

Code	Pasta Formula (%)			Production Parameters	
	Semolina	FF	FC	Pressure (MPa)	Line Output (kg h^−1^)
CON	100	-	-	12.0	23.0 ^a^ ± 0.1
FF5	95	5		10.5	22.2 ^bdac^ ± 0.6
FF9	91	9		0.95	22.4 ^bac^ ± 0.4
FF13	87	13		0.92	22.2 ^bdac^ ± 0.8
FF17	83	17		0.83	22.7 ^ba^ ± 0.3
FF20	80	20		0.82	22.5 ^ba^ ± 0.1
FF23	77	23		0.82	21.5 ^dc^ ± 0.2
FC5	100		5	0.99	22.5 ^ba^ ± 0.2
FC 9	95		9	0.95	21.9 ^bdc^ ± 0.3
FC 13	91		13	0.9	21.8 ^bdc^ ± 0.0
FC 17	87		17	0.82	21.7 ^bdc^ ± 0.3
FC 20	83		20	0.8	21.8 ^bdc^ ± 0.3
FC 23	80		23	0.8	21.3 ^d^ ± 0.8

Explanation: CON–control (100% semolina); FF–flaxseed flour; FC–flaxseed cake; data are presented as mean ± standard deviation; data value of each parameter with different superscript letter in rows are significantly different (Duncan test, *p* ≤ 0.05).

**Table 2:** Variation of color parameters of cooked pasta with flaxseed flour (FF) and flaxseed cake (FC).

Sample	L *	a *	b *	∆E *	WI
CON	61.1 ^a^ ± 1.9	1.3 ^f^ ± 0.3	20.5 ^a^ ± 1.6	-	55.9 ^a^ ± 1
FF5	49.2 ^b^ ± 1.3	4.6 ^bac^ ± 0.5	11.8 ^b^ ± 1.3	15.2 ^g^ ± 1.6	47.6 ^b^ ± 1
FF9	46.7 ^c^ ± 0.7	5.3 ^a^ ± 0.4	8.8 ^c^ ± 0.7	19.0 ^f^ ± 0.8	45.7 ^cb^ ± 0.6
FF13	45.5 ^dc^ ± 1.2	4.9 ^bac^ ± 0.4	6.3 ^ed^ ± 0.7	21.5 ^e^ ± 0.9	44.9 ^cd^ ± 1.2
FF17	43.2 ^dfe^ ± 0.5	4.7 ^bac^ ± 0.7	5.0 ^efg^ ± 0.9	24.0 ^cd^ ± 0.4	42.8 ^fde^ ± 0.6
FF20	42.3 ^fe^ ± 1.1	4.3 ^bdc^ ± 0.3	3.8 ^hig^ ± 0.4	25.4 ^cb^ ± 0.9	42.0 ^fe^ ± 1.1
FF23	37.4 ^g^ ± 3.9	4.1 ^bc^ ± 0,4	3.6 ^hig^ ± 0.5	29.3 ^a^ ± 2.8	37.2 ^g^ ± 4
FC5	46.6 ^c^ ± 2.9	4.3 ^bdc^ ± 0.9	12 ^b^ ± 2.6	17.4 ^f^ ± 2.2	45.0 ^cd^ ± 3.2
FC 9	44.9 ^dc^ ± 0.6	4.6 ^bac^ ± 0.4	7.3 ^d^ ± 0.7	21.1 ^e^ ± 0.7	44.3 ^cde^ ± 0.6
FC 13	44.5 ^dce^ ± 1.2	4.5 ^bc^ ± 0.4	5.4 ^ef^ ± 0.6	22.7 ^ed^ ± 0.6	44.1 ^cde^ ± 1.2
FC 17	43.4 ^dfe^ ± 1.2	3.7 ^ed^ ± 0.2	3.2 ^hi^ ± 0.3	26.4 ^cb^ ± 0.8	43.2 ^cfde^ ± 1.2
FC 20	42.0 ^fe^ ± 0.9	3.2 ^e^ ± 0.3	2.4 ^i^ ± 0.4	24.9 ^b^ ± 0.8	41.9 ^fe^ ± 0.9
FC 23	41.4 ^f^ ± 2.0	3.1 ^e^ ± 0.6	2.1 ^i^ ± 0.8	27.1 ^cb^ ± 1.4	41.3 ^fe^ ± 0.9

Explanation: * concern CIE–lab color scale, ΔE*–the total color difference, WI–the whiteness index, data are presented as mean ± standard deviation. Data value of each parameter with different superscript letter in rows are significantly different (Duncan test, *p* ≤ 0.05).

**Table 3:** The cooking properties of pasta with flaxseed flour (FF) and flaxseed cake (FC).

Sample	Minimum Cooking Time (min)	Cooking Losses (% d.b.)	Weight Increase Index (-)	Volume Increase Index (-)
CON	5 ^e^ ± 0.15	5.67 ^a^ ± 0.25	2.12 ^b^ ± 0.11	2.66 ^b^ ± 0.12
FF5	6.05 ^cd^ ± 0.09	5.41 ^b^ ± 0.05	2.18 ^ab^ ± 0.08	2.73 ^b^ ± 0.14
FF9	6.42 ^cb^ ± 0.06	4.94 ^d^ ± 0.03	2.24 ^ab^ ± 0.1	2.75 ^b^ ± 0.1
FF13	6.45 ^b^ ± 0.05	4.78 ^d^ ± 0.1	2.27 ^ab^ ± 0.09	2.76 ^b^ ± 0.11
FF17	7.05 ^a^ ± 0.09	4.5 ^e^ ± 0.12	2.27 ^ab^ ± 0.07	2.81 ^b^ ± 0.14
FF20	7.1 ^a^ ± 0.09	4.23 ^f^ ± 0.04	2.29 ^ab^ ± 0.06	2.84 ^b^ ± 0.13
FF23	6.92 ^a^ ± 0.04	3.99 ^g^ ± 0.01	2.35 ^a^ ± 0.07	2.88 ^b^ ± 0.13
FC5	5.82 ^d^ ± 0.32	5.25 ^bc^ ± 0.13	2.17 ^ab^ ± 0.05	2.86 ^b^ ± 0.17
FC 9	5.82 ^d^ ± 0.32	5.15 ^c^ ± 0.05	2.19 ^ab^ ± 0.05	2.94 ^b^ ± 0.15
FC 13	5.87 ^d^ ± 0.37	5.14 ^c^ ± 0.11	2.23 ^ab^ ± 0.07	3.3 ^a^ ± 0.13
FC 17	6.05 ^cd^ ± 0.09	4.86 ^d^ ± 0.12	2.25 ^ab^ ± 0.11	3.32 ^a^ ± 0.17
FC 20	6.1 ^bcd^ ± 0.09	4.75 ^d^ ± 0.16	2.28 ^ab^ ± 0.09	3.34 ^a^ ± 0.1
FC 23	6.1 ^bcd^ ± 0.09	4.44 ^e^ ± 0.06	2.3 ^ab^ ± 0.06	3.37 ^a^ ± 0.1

Explanation: data are presented as mean ± standard deviation, data value of each parameter with different superscript letter in rows are significantly different (Duncan test, *p* ≤ 0.05).

**Table 4:** The strength test for pasta with flaxseed flour (FF) and flaxseed cake (FC).

Sample	Cutting Force Firmness (N)	Sample	Cutting Force Firmness (N)
CON	1.03 ^a^ ± 0.02	CON	1.03 ^a^ ± 0.02
FF5	0.94 ^d^ ± 0.00	FC5	1.03 ^a^ ± 0.05
FF9	0.92 ^de^ ± 0.00	FC 9	1.03 ^a^ ± 0.03
FF13	0.91 ^e^ ± 0.01	FC 13	1.00 ^b^ ± 0.02
FF17	0.92 ^ed^ ± 0.03	FC 17	0.98 ^bc^ ± 0.02
FF20	0.91 ^e^ ± 0.01	FC 20	0.97 ^c^ ± 0.04
FF23	0.92 ^ed^ ± 0.01	FC 23	0.89 ^f^ ± 0.00

Explanation: data are presented as mean ± standard deviation. Data value of each parameter with different superscript letter in rows are significantly different (Duncan test, *p* ≤ 0.05).

**Table 5:** Chemical composition [% d.b.] and energy value [kcal/100g] of raw materials and pasta with flaxseed flour (FF) and flaxseed cake (FC).

Sample	Dry Weight	Ash	Protein	Fat	IDF	SDF	TDF	Carbohydrates	Energy Value
Sem	88.54 ^a^ ± 0.31	0.91c ± 0.01	13.36 ^c^ ± 0.07	0.49 ^c^ ± 0.01	1.81 ^c^ ± 0.08	2.73 ^b^ ± 0.15	4.53 ^c^ ± 0.07	69.25 ^a^ ± 0.46	343.9 ^b^ ± 1.3
FF	92.95 ^b^ ± 0.12	4.94 ^b^ ± 0.01	33.32 ^b^ ± 0.17	15.27 ^a^ ± 0.04	16.97 ^b^ ± 0.23	12.62 ^a^ ± 0.91	29.59 ^b^ ± 0.14	9.83 ^b^ ± 0.81	369.2 ^a^ ± 2
FC	92.84 ^b^ ± 0.04	6.07 ^a^ ± 0.04	34.97 ^a^ ± 0.13	12.41 ^b^ ± 0.08	17.97 ^a^ ± 0.25	14.48 ^a^ ± 0.44	32.45 ^a^ ± 0.18	6.94 ^c^ ± 0.1	344.2 ^b^ ± 0.5
CON	89.05 ^b^ ± 0.1	0.96 ^h^ ± 0.01	13.37 ^g^ ± 0.04	0.5 ^i^ ± 0.02	1.71 ^g^ ± 0.25	2.84 ^d^ ± 0.3	4.55 ^f^ ± 0.56	69.67 ^a^ ± 0.4	345.8 ^a^ ± 0.9
FF5	90.5 ^a^ ± 0.11	1.11 ^g^ ± 0.08	14 ^f^ ± 0.06	0.73 ^h^ ± 0.03	2.75 ^fg^ ± 0.33	3.55 ^cd^ ± 0.81	6.29 ^ef^ ± 1.14	68.36 ^ab^ ± 1.28	348.6 ^a^ ± 2.3
FF9	90.18 ^ab^ ± 0.13	1.31 ^ef^ ± 0.02	14.72 ^e^ ± 0.02	0.87 ^g^ ± 0.01	3.2 ^ef^ ± 0.14	3.75 ^bcd^ ± 0.84	6.95 ^def^ ± 0.98	66.33 ^b^ ± 0.81	345.9 ^a^ ± 1.4
FF13	89.87 ^ab^ ± 0.7	1.41 ^e^ ± 0.06	15.84 ^d^ ± 0.13	1.23 ^f^ ± 0.01	3.94 ^cdef^ ± 0.79	4.23 ^abcd^ ± 0.25	8.17 ^cde^ ± 1.04	63.21 ^cd^ ± 1.94	343.6 ^a^ ± 5.1
FF17	90.24 ^ab^ ± 0.04	1.56 ^d^ ± 0.02	16.42 ^c^ ± 0.03	1.45 ^e^ ± 0.07	4.53 ^bcd^ ± 0.89	4.42 ^abcd^ ± 0.02	8.95 ^abcde^ ± 0.87	61.86 ^de^ ± 0.87	344.1 ^a^ ± 2.2
FF20	90.73 ^a^ ± 0.81	1.71 ^c^ ± 0.03	17.19 ^b^ ± 0.18	1.59 ^d^ ± 0.02	4.97 ^abc^ ± 0.07	5.36 ^abc^ ± 0.62	10.34 ^abc^ ± 0.7	59.9 ^ef^ ± 1.67	343.4 ^a^ ± 4.4
FF23	90.03 ^ab^ ± 1.33	1.9 ^b^ ± 0.11	17.93 ^a^ ± 0.27	3.05 ^a^ ± 0.13	5.56 ^ab^ ± 0.09	5.87 ^a^ ± 2.45	11.44 ^ab^ ± 2.54	56.65 ^g^ ± 1.99	348.7 ^a^ ± 5.1
FC5	90.04 ^ab^ ± 0.05	1.25 ^f^ ± 0.03	14.14 ^f^ ± 0.03	0.38 ^j^ ± 0.03	3.67 ^cdef^ ± 0.87	3.52 ^cd^ ± 0.51	7.19 ^def^ ± 1.37	67.08 ^ab^ ± 1.45	342.7 ^a^ ± 3.2
FC9	90.46 ^a^ ± 0.35	1.39 ^e^ ± 0.05	14.93 ^e^ ± 0.11	0.8 ^gh^ ± 0.03	3.57 ^def^ ± 0.23	3.99 ^abcd^ ± 0.42	7.56 ^cde^ ± 0.65	65.78 ^bc^ ± 0.11	345.1 ^a^ ± 0.1
FC13	90.44 ^a^ ± 0.23	1.57 ^d^ ± 0.04	15.72 ^d^ ± 0.01	1.14 ^f^ ± 0.04	4.24 ^cde^ ± 0.81	4.5 ^abcd^ ± 0.19	8.74 ^bcde^ ± 1	63.28 ^cd^ ± 1.33	343.8 ^a^ ± 2.9
FC17	90.28 ^ab^ ± 0.72	1.73 ^c^ ± 0.02	16.51 ^c^ ± 0.05	1.84 ^c^ ± 0.02	4.62 ^bcd^ ± 0.4	4.75 ^abcd^ ± 0.61	9.36 ^abcd^ ± 1.01	60.84 ^def^ ± 1.78	344.7 ^a^ ± 4.7
FC20	90.75 ^a^ ± 0.03	1.93 ^ab^ ± 0.02	17.16 ^b^ ± 0.01	2.15 ^b^ ± 0.06	5.64 ^ab^ ± 0.51	5.58 ^abc^ ± 0.45	11.22 ^ab^ ± 0.96	58.29 ^fg^ ± 0.84	343.6 ^a^ ± 2
FC23	91.04 ^a^ ± 0.14	2.01 ^a^ ± 0.01	18.06 ^a^ ± 0.06	3.08 ^a^ ± 0.06	5.95 ^a^ ± 0.61	5.74 ^ab^ ± 0.51	11.69 ^a^ ± 1.11	56.2 ^g^ ± 1.36	348.1 ^a^ ± 2.4

Explanation: IDF–insoluble dietary fiber. SDF–soluble dietary fiber. TDF–total dietary fiber; Data are presented as mean ± standard deviation, data value of each parameter with different superscript letter in rows are significantly different (Duncan test. *p* ≤ 0.05).
